# Functional Differences between Proteasome Subtypes

**DOI:** 10.3390/cells11030421

**Published:** 2022-01-26

**Authors:** Joanna Abi Habib, Julie Lesenfants, Nathalie Vigneron, Benoit J. Van den Eynde

**Affiliations:** 1 Ludwig Institute for Cancer Research, 1200 Brussels, Belgium; joanna.abi-habib@bru.licr.org (J.A.H.); julie.lesenfants@bru.licr.org (J.L.); 2 De Duve Institute, Université Catholique de Louvain, 1200 Brussels, Belgium; 3 Walloon Excellence in Life Sciences and Biotechnology (WELBIO), 1200 Brussels, Belgium; 4 Ludwig Institute for Cancer Research, Nuffield Department of Medicine, University of Oxford, Oxford OX3 7DQ, UK

**Keywords:** proteasome subtypes, protein degradation, ATP- and ubiquitin-dependent degradation, ATP- and ubiquitin-independent degradation, MHC class I peptides, autoimmune diseases

## Abstract

Four proteasome subtypes are commonly present in mammalian tissues: standard proteasomes, which contain the standard catalytic subunits β1, β2 and β5; immunoproteasomes containing the immuno-subunits β1i, β2i and β5i; and two intermediate proteasomes, containing a mix of standard and immuno-subunits. Recent studies revealed the expression of two tissue-specific proteasome subtypes in cortical thymic epithelial cells and in testes: thymoproteasomes and spermatoproteasomes. In this review, we describe the mechanisms that enable the ATP- and ubiquitin-dependent as well as the ATP- and ubiquitin-independent degradation of proteins by the proteasome. We focus on understanding the role of the different proteasome subtypes in maintaining protein homeostasis in normal physiological conditions through the ATP- and ubiquitin-dependent degradation of proteins. Additionally, we discuss the role of each proteasome subtype in the ATP- and ubiquitin-independent degradation of disordered proteins. We also discuss the role of the proteasome in the generation of peptides presented by MHC class I molecules and the implication of having different proteasome subtypes for the peptide repertoire presented at the cell surface. Finally, we discuss the role of the immunoproteasome in immune cells and its modulation as a potential therapy for autoimmune diseases.

## 1. Introduction

Protein turnover is essential to maintain appropriate levels of proteins inside cells and to maintain cellular integrity. A major enzyme involved in regulating protein turnover is the proteasome, a large multi-catalytic protease complex that specifically degrades undesirable or damaged proteins [1]. In the course of protein degradation, the proteasome generates peptides of about 3 to 22 amino acids [2], which can be further degraded by cytosolic peptidases to recycle the pool of amino acids. Some of the peptides produced by the proteasome escape the additional degradation steps and are transferred into the endoplasmic reticulum (ER) to be loaded onto major histocompatibility complex (MHC) class I molecules and displayed at the cell surface [3]. In healthy cells, peptides loaded on MHC class I originate from normal autologous proteins and thus fail to activate the immune system. However, in infected and transformed cells, some of the peptides presented by MHC class I are derived from viral or tumour-associated proteins and can, therefore, activate cytolytic T lymphocytes (CTL), thus helping the body to eliminate the abnormal cells [3,4]. These essential cellular functions make the proteasome a guardian of proteome integrity and a key element in the immunological screening for infected or cancer cells.

## 2. The Proteasome Structure

### 2.1. Structure of the 20S Proteasome

**Figure 1:**
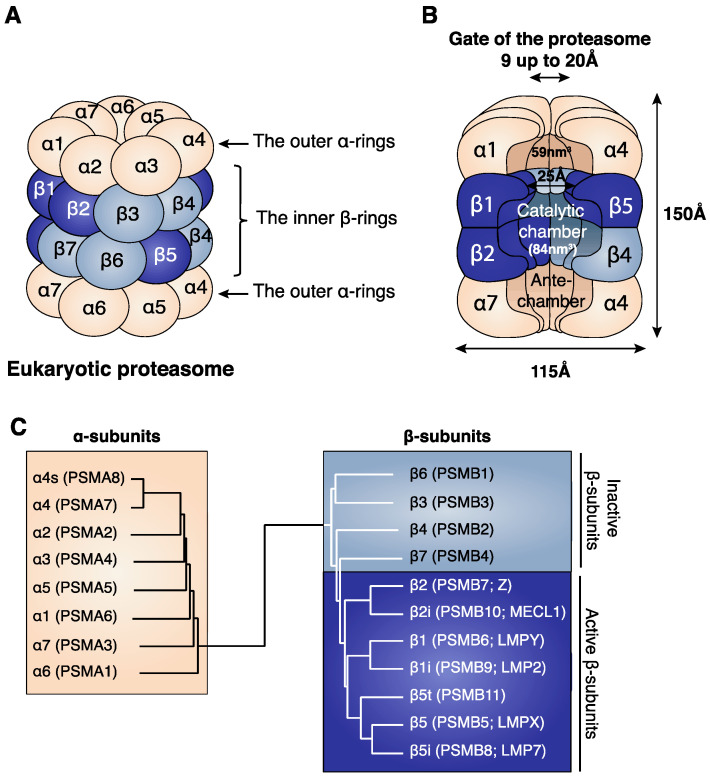
(**A**) Structure of the eukaryotic proteasome. The identical α-rings are each composed of seven distinct α-subunits (α1–α7) (beige). The identical inner β-rings are each composed of seven distinct β-subunits (β1–β7). Only three of the seven β-subunits are catalytically active, and these are β1, β2 and β5 (dark blue). (**B**) Cross section of the 20S proteasome showing its internal cavities: the two antechambers of 59 nm^3^ and the central catalytic chamber of 84 nm^3^, where protein degradation takes place [9]. The length of the eukaryotic proteasome is about 150 Å and its diameter is about 115 Å [8]. The gate of the proteasome, which is delimited by the outer α-rings, has a pore size ranging from 9 to 13 Å in its closed conformation and of 20 Å in its open conformation [5,10]. (**C**) Relationship between the α- and β-subunits. Dendrogram showing the similarities between the eukaryotic α-subunits and between the eukaryotic β-subunits. The names of the proteasome subunits are mentioned following the common nomenclature (α and β). In parentheses we added the HUGO (human genome organization) nomenclature (PSMA and PSMB), as well as a previously common nomenclature for the catalytic subunits β1 (LMPY), β2 (Z), β5 (LMPX), β1i (LMP2), β2i (MECL1) and β5i (LMP7).

Our knowledge of proteasome structure originates from X-ray crystallographic studies, which were initially performed on proteasomes isolated from archaebacterium *Thermoplasma acidophilum* (PDB: 1PMA) [5] and were later followed by the structural analysis of proteasomes isolated from numerous eukaryotic cells [6,7,8] (yeast proteasome, PDB: 1RYP [6]; murine proteasomes, PDB: 3UNE and 3UNH [7]; and bovine proteasome, PDB: 1IRU [8]). The core of the proteasome, called the 20S proteasome, is conserved among organisms and consists of a barrel-shaped particle made of four stacked heptameric rings. The two identical outer α-rings are composed of seven α-subunits, while the two identical inner β-rings contain seven β-subunits (Figure 1A) [5,6,8].

These rings delimit three interconnected cavities, namely the two antechambers formed by the adjacent α and β rings and the central catalytic chamber, which is delimited by the two inner β-rings and inside which proteins are degraded (Figure 1B) [5,9]. The active sites of the proteasome catalytic subunits are sequestered inside the catalytic chamber, thereby preventing uncontrolled degradation of the surrounding cytoplasmic proteins. Proteins targeted for proteasome degradation need to be unfolded to penetrate the gates formed by the two outer α-rings of the 20S proteasome, reach the antechamber and finally access the catalytic chamber (Figure 1B) [5,6,11,12].

From archaea to mammals, the 20S proteasome subunits have acquired a considerable level of diversity. Archaebacterial proteasomes are composed of 14 copies of two different subunits: the inactive α-subunit and the catalytically active β-subunit. Together these two subunits assemble into four stacked homo-heptameric rings, thus forming the conserved barrel-shaped structure of 20S proteasomes [5]. Eukaryotic proteasomes consist of two copies of 14 different subunits. Based on their sequence similarities (Figure 1C) and their location in the 20S proteasome, these 14 subunits are classified into seven-α type subunits (α1–α7) and seven β-type subunits (β1–β7), three of which are catalytically active: β1 (PSMB6 or LMPY), β2 (PSMB7 or Z) and β5 (PSMB5 or LMPX) (Figure 1A) [6,9]. In addition to the seven standard β-subunits, mammalian cells encode four additional active β-subunits: the three immuno-subunits β1i (PSMB9 or LMP2), β2i (PSMB10 or MECL1) and β5i (PSMB8 or LMP7), and the thymus-specific subunit β5t (PSMB11) [13,14]. All β-subunits except β3 and β4 contain a pro-peptide sequence. Some of these pro-peptides, namely the pro-peptides of the subunits β2, β5 and β5i, play an important role in proteasome assembly [15,16]. The pro-peptides of the catalytic subunits are autolyzed between a glycine residue and a threonine residue following the correct assembly of the 20S proteasome. This results in the production of the mature catalytic subunits, having the conserved active threonine at position 1 (Thr1) [16,17]. The pro-peptides of the β6 and β7 subunits are also removed through the action of one of the catalytically active subunits found in the 20S proteasome [18]. One additional inactive α-subunit can be detected in mammalian testes, the α4s subunit (PSMA8) [19]. In addition to its expression in testes, protein atlas data show that PSMA8 is expressed at low levels in immunological tissues, such as tonsils and lymph nodes. These data require further investigations. Based on their sequence similarities, some mammalian proteasome subunits are highly similar and can be grouped in the same category (Figure 1C):

- The two homologous subunits α4 (PSMA7) and α4s (PSMA8).
- The two homologous subunits β1 (PSMB6) and β1i (PSMB9).
- The two homologous subunits β2 (PSMB7) and β2i (PSMB10).
- The three homologous subunits β5 (PSMB5), β5i (PSMB8) and β5t (PSMB11).

Subunits belonging to the same category do not coexist in the same ring, which always obeys an α1–α7/β1–β7 heptameric structure. The diversity in the mammalian proteasome subunits results in the production of different proteasome subtypes, which are described below. Of note, although no study has demonstrated the coexistence of homologous subunits belonging to the same category in one 20S proteasome particle, the existence of 20S proteasome particles composed of two rings each containing different homologous subunits cannot be excluded.

### 2.2. Proteasome Subtypes

#### 2.2.1. The Standard Proteasome

**Figure 2:**
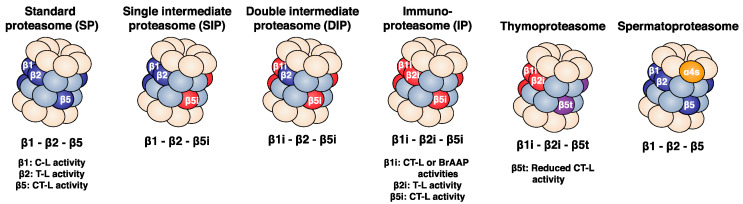
There are six proteasome subtypes that differ in their subunit composition. The standard proteasome (SP) contains the constitutive catalytic subunits β1, β2 and β5, while the immunoproteasome (IP) contains the immuno-subunits β1i, β2i and β5i. The intermediate proteasomes comprise a mixed assortment of constitutive and inducible subunits: the single intermediate proteasome (SIP) contains β1, β2 and β5i, while the double intermediate proteasome (DIP) contains β1i, β2 and β5i. The thymoproteasome contains catalytic subunit β5t, which is homologous to β5 and β5i, along with subunits β1i and β2i. Each catalytic subunit is characterized by different cleavage specificities as indicated in the lower part of the figure. Finally, the spermatoproteasome contains an α4s subunit instead of the standard α4 and expresses, in its catalytic chamber, the same assortment of catalytic subunits as the SP. Abbreviations used: caspase-like (C-L), trypsin-like (T-L), chymotrypsin-like (CT-L), and branched amino-acid preferring activity (BrAAP).

The standard proteasome (SP) is expressed in all eukaryotic cells and is essential to maintain protein homeostasis [20]. The SP expresses all the constitutive proteasome subunits, which include α4, β1, β2 and β5 (Figure 2). In mammalian cells, constitutive expression of the genes encoding the SP subunits is regulated by numerous transcription factors. The nuclear transcription factor (NF)-Y regulates the expression of α2 (PSMA2), α5 (PSMA5), α7 (PSMA3), β3 (PSMB3), β4 (PSMB2) and β6 (PSMB1) [21]. The signal transducer and activator of transcription (STAT)-3 regulates the expression of all β-subunits [22]. Additionally, in response to proteasome inhibition and growth signals, the activity of the nuclear factor erythroid-derived 2-related factor 1 (Nrf1) is increased, leading to higher expression of the genes that encode all the SP subunits [23,24,25]. Finally, in oxidative stress conditions, the interaction between Nrf2 and KEAP1 is disrupted, inducing the translocation of Nrf2 into the nucleus. Once in the nucleus, Nrf2 binds the antioxidant response element (ARE) found in the promoter region of all the genes encoding proteasome subunits, thus increasing the expression of these genes [26,27,28].

Three major catalytic activities are associated to the proteasome: caspase-like, trypsin-like and chymotrypsin-like activities, which cleave after acidic, basic and hydrophobic residues, and whose activity is generally linked to β1, β2 and β5, respectively [29,30,31]. These cleavage specificities are mostly the result of the surface properties of the S1 substrate binding pocket, which harbours the active Thr1. The S1 pocket is found in all catalytic subunits and interacts with the P1 amino acid, which is the denomination given to the amino acid located upstream from the cleavage site. The crystal structures of yeast, bovine and murine SP revealed a positively charged arginine located at position 45 on the bottom of the mature β1 subunit, which is well suited for cleaving after negatively charged residues (caspase-like activity) (PDB: 3E47 [32]) [7,8,32]. Moreover, the structural analysis of the bovine and murine mature β2 subunit revealed the presence of a glycine at position 45 and a negatively charged aspartate at position 53 also found at the bottom of the S1 pocket, in line with the fact that β2 cleaves after positively charged residues (trypsin-like activity) [7,8]. In yeast, the mature β2 has a negatively charged glutamate at position 53 instead of an aspartate. This substitution does not change the surface property of the S1 pocket of the yeast β2 subunit as compared to the mammalian β2 subunit [32]. Finally, the yeast, bovine and murine mature β5 subunit showed a hydrophobic methionine at position 45, in line with a cleaving activity after hydrophobic residues (chymotrypsin-like activity) (Figure 2) [7,8,32]. Other activities were also associated with the β5 subunit, such as branched amino-acid preferring activity (BrAAP) and small neutral amino acid preferring activity (SNAAP) [18].

#### 2.2.2. The Immunoproteasome

The immunoproteasome (IP) is constitutively expressed in a broad range of immune cells, such as T cells, B cells and antigen presenting cells [20]. Its expression can be induced by proinflammatory cytokines, such as interferon (IFN) γ, type I IFNs and tumour necrosis factor (TNF) α [33,34], or in response to LPS [35] or to stress conditions [36]. In the catalytic chamber of the IP, the three catalytic subunits β1, β2 and β5 are replaced by catalytic immuno-subunits β1i, β2i and β5i, respectively (Figure 2) [37,38,39,40,41,42,43,44]. In humans, the genes encoding β1i and β5i are located on chromosome 6 and clustered in the MHC class II region with the genes encoding transporter associated with antigen processing (TAP)-1 and TAP-2 [41,42]. The gene encoding subunit β2i is found on chromosome 16. The promoters of all three immuno-subunit genes contain binding sites for IFN regulatory factor (IRF)-1 transcription factor, conferring responsiveness to IFNγ [45,46,47]. Furthermore, their promoter regions also display binding sites for the nuclear factor kappa B (NF κB) or the cAMP response element binding protein (CREB), which triggers the expression of these inducible subunits after TNF α stimulation or activation of the nitric oxide (NO^•^) signalling pathway, respectively [48,49,50]. Finally, although all three immuno-subunits are induced in response to oxidative stress, Nrf2 only partially regulates this expression. As a matter of fact, the β5i subunit is the only immuno-subunit that has an ARE element in its promoter region [28].

The replacement of the standard catalytic subunits by immuno-subunits increases the chymotrypsin-like and trypsin-like activities of the proteasome while lowering its caspase-like activity (Figure 2) [33,51,52]. Analysis of the crystal structure of the murine SP and IP revealed differences between homologous subunits that likely explain their cleavage specificities. Two substitutions were observed in the S1 pocket of β1i when compared to β1: R45L and T31F. These substitutions favour the binding of small hydrophobic or branched P1 residues instead of acidic residues (chymotrypsin-like or BRAAP activities instead of caspase-like) (Figure 2) [7]. A recent cryo-electron microscopy (cryo-EM) analysis on bovine IP confirmed that the T31F substitution affects the surface property of the S1 pocket of β1i, contributing to a lower caspase-like activity of β1i. A conformational rearrangement of the C-terminal tail and the linker region between Gly133 and Leu139 of β1i compared to β1 might also play a role in the functional differences between the two homologous subunits (PDB: 7DR7) [53]. In addition, analysis of the crystal structure of murine SP and IP revealed a difference in the size of the S1 pocket between β5 and β5i: a S53Q substitution in β5i promotes strong van der Waals interactions between M45 and Q53, widening the S1 pocket of the β5i subunit, making it significantly larger than the S1 pocket of β5. As a result, the S1 pocket of β5i can accommodate large hydrophobic residues. Finally, the S1 pocket of β2 and β2i are identical, except for a D53E substitution. This substitution does not change the surface property of the β2i S1 pocket as compared to β2 [7]. Cryo-EM analysis of bovine IP showed, however, that the C-terminal loop of β2i exhibited a minor outward shift when compared to the C-terminal loop of β2. This conformational change might play a role in the functional differences between the β2 and the β2i subunits [53].

#### 2.2.3. The Intermediate Proteasomes

Over a decade ago, two additional subtypes of proteasome, which are intermediate between the SP and the IP, were identified in healthy tissues, in tumour cell lines and in normal cell lines. These two proteasomes are the single intermediate proteasome (SIP), containing β1, β2 and β5i; and the double intermediate proteasome (DIP), containing subunit β2 along with β1i and β5i (Figure 2). Production of these intermediate proteasomes is facilitated by the rules of cooperative incorporation of immuno-subunits into nascent proteasomes. Indeed, the incorporation of β2i is facilitated by the presence of β1i, and its maturation requires the activity of β5i. Moreover, β1i is the first subunit incorporated to the newly formed β-ring, and its maturation also requires the activity of β5i [54,55]. Finally, β5i can incorporate a nascent proteasome without the presence of the other immuno-subunits. Despite these rules of cooperative incorporation, studies using mice that are KO for one immuno-subunit, notably β1i or β5i, suggest that other intermediate proteasomes could exist [56,57]. Indeed, retinal lysates of β5i-deficient mice contain proteasomes expressing β1i and β5 [57]. Moreover, B cells of β1i-deficient mice contain proteasomes expressing β2i and β5i along with β1 [56]. Beta5i-deficient T2 cells were shown to contain low amounts of intermediate proteasome containing β1, β2i and β5 [55]. Although such proteasomes exist in these artificial systems, their presence in normal tissues and tumours has not been observed thus far.

Initially, the existence intermediate proteasomes was suggested by the fact that only one or two immuno-subunits were detected in some tissues [58]. It is the use of antibodies able to specifically recognize immuno-subunits in their native conformation that made it possible to determine the exact stoichiometry of intermediate proteasomes and to evaluate their presence in liver, kidney, small bowel, colon, tumour, and dendritic cells [59]. Moreover, it was recently shown that the SIP is expressed in pancreatic β-cells and that β1i and β5i are upregulated by a low concentration of IL-1β [60]. Studies of the catalytic activity of the two intermediate proteasomes in native conditions showed that both SIP and DIP display chymotrypsin-like and trypsin-like activities that are intermediate between the SP and the IP. Finally, the SIP displays a caspase-like (cleavage after acidic amino acids) activity similar to that of the SP, while this activity is low in the DIP. This is because the caspase-like activity is assigned to the β1 subunit, which is present in both SP and SIP [31,59,61].

#### 2.2.4. The Thymoproteasome

The thymoproteasome is an additional proteasome subtype that is exclusively expressed in cortical thymic epithelial cells (cTECs), which mediate the positive selection of progenitor T cells in the thymus [14,62]. The catalytic chamber of the thymoproteasome contains the two immuno-subunits β1i and β2i assembled with β5t (Figure 2), which is homologous to β5 and β5i (Figure 1C) and is specifically expressed by cTECs [14]. Expression of this active β-subunit is transcriptionally regulated by Foxn1, a transcription factor responsible for the specific expression of β5t in cTECs [63,64,65]. The incorporation of β5t instead of β5 or β5i reduces the chymotrypsin-like activity of the thymoproteasome dramatically as compared to the SP and the IP. As opposed to the hydrophobic S1 substrate binding pocket of both β5 and β5i, the S1 pocket of β5t has many hydrophilic residues that reduce its ability to interact with hydrophobic P1 residues [14].

#### 2.2.5. The Spermatoproteasome

Unlike the five proteasome subtypes described above, the spermatoproteasome has a unique combination of α-subunits. The catalytic chamber of the spermatoproteasome is identical to that of the SP; however, the difference lies in the α-ring that contains an alternative α4 subunit, the α4s (or PSMA8) (Figure 2) [66,67]. This subunit does not modify the catalytic activities of the proteasome and is found in mammalian testes more precisely, in male germ cells [19]. This newly identified proteasome plays a key role in spermatogenesis [67,68,69]. The α4s subunit is required for the proper formation of proteasomes in testes, for the acetylation-dependent degradation of core histones in spermatocytes and for the proper progression of meiosis in male germ cells [67,68,69].

### 2.3. Regulatory Particles

The different proteasome subtypes are found in mammalian cells either as free 20S proteasomes or associated to regulatory particles (RP). The interaction of RP with the α-rings of the 20S proteasome facilitates the opening of the gate and controls the entry of substrates into the catalytic chamber of the proteasome.

#### 2.3.1. The 19S Regulatory Particle

**Figure 3:**
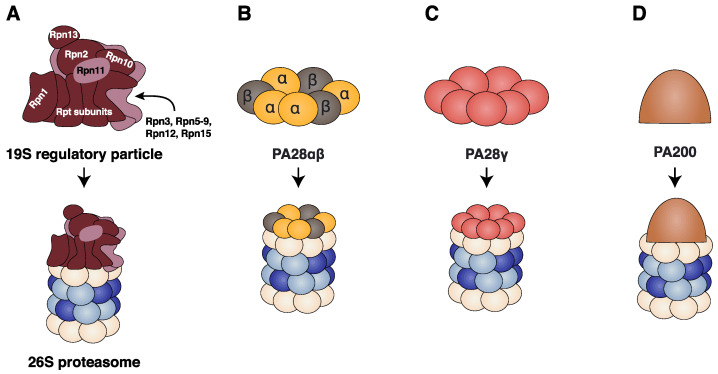
Proteasome regulators. (**A**) 19S regulator particle harbours two subcomplexes: the lid subcomplex contains nine subunits (Rpn3, Rpn5–9, Rpn11, Rpn12 and Rpn15; light brown) and the base subcomplex contains ten subunits (dark brown): Rpn2, Rpn1, Rpn10, Rpn13 and six distinct Rpt1-Rpt6 (regulatory particle ATPase subunits) that form the AAA+ motor of the 19S regulatory particle. The 19S regulator associates to the 20S proteasome to form the 26S proteasome. (**B**) The PA28αβ regulator is a ring-shaped hetero-heptameric ring composed of four PA28α subunits and three PA28β subunits. (**C**) The PA28γ regulator is a ring-shaped homo-heptameric ring composed of seven PA28γ subunits. (**D**) The PA200 regulator is a 200 kDa monomeric regulator. All four regulatory particles can bind to one or both α-rings of the 20S proteasome.

The 19S regulator (or Proteasome Activator (PA)-700) is a multimeric protein complex that can bind to one or both α-rings of the 20S proteasome to form the 26S proteasome (Figure 3A). The function of the 19S regulator is to recognize ubiquitinated proteins, remove their ubiquitin tag, unfold and translocate them into the 20S proteasome chamber where they are degraded. The 19S RP is composed of at least 19 subunits assembled into the lid and the base subcomplexes. The lid subcomplex comprises nine Rpn subunits (Rpn3, Rpn5–9, Rpn11, Rpn12 and Rpn15) and acts as a scaffold supporting one side of the base subcomplex (Figure 3A) [70,71]. The base subcomplex consists of ten subunits: Rpn2, the three ubiquitin receptors Rpn1, Rpn10 and Rpn13 and six distinct ATPase subunits Rpt1–Rpt6 (regulatory particle ATPase subunits) that form the ring-shaped hetero-hexameric motor of the 19S RP (Figure 3A) [72,73,74,75,76]. Each Rpt consists of an N-terminal α-helix, an oligonucleotide-binding (OB)-fold domain, an AAA+ (ATPases associated with diverse cellular activities) domain with a conserved loop (called hereafter a pore loop). At the C-terminus of Rpt2, Rpt3 and Rpt5 a conserved HbYX (‘Hb’ hydrophobic residue, ‘Y’ tyrosine and ‘X’ any amino acid) motif is found. The pore loops of the AAA+ domains project into the centre of the hexameric ring and sterically interact with the protein substrate [10,70,71,77].

#### 2.3.2. The PA28αβ Regulator

The stimulation of cells with IFNγ induces not only the expression of the immuno-subunits (β1i, β2i and β5i) but also the expression of PA28α and PA28β, whose encoding genes are found in the MHC class II region of chromosome 6 [78,79]. These genes encode two homologous 28kDa subunits, which assemble into a ring-shaped hetero-heptameric complex that binds one or two α-rings of the 20S proteasome and stimulates the degradation of short peptides but not full-length folded proteins (Figure 3B) [80,81,82,83,84]. Furthermore, the PA28αβ regulator can bind to the free α-ring of a 26S proteasome to form a hybrid proteasome [85]. A recent crystallographic study performed on the PA28αβ complex indicated that it is composed of four PA28α and three PA28β subunits (Figure 3B) (PDB: 5MSJ, 5MSK and 5MX5) [86]. The overall structures of PA28α and PA28β are similar and consist of four long helices of 33–45 residues with a highly conserved linker domain between helices 2 and 3 called the “activation loop” (PDB: 1AVO) [87]. A recent study of mammalian PA28αβ-IP at near atomic resolution by cryo-EM revealed that the C-terminal tails of four consecutive PA28αβ subunits insert into the α-ring pockets of the IP, namely the pockets of α1/2, α2/3, α3/4 and α4/5. These insertions stabilize the interaction between PA28αβ and the IP and cause the leaning of the regulator towards the α3-α4 side of the 20S proteasome. This position facilitates the interaction between the activation loops of the PA28αβ and the reverse turn of the α-subunits implicated in the gate formation of the 20S proteasome (α2, α3 and α4). The interactions between the activation loops and the reverse turns disturb the allosteric structure of the gate resulting in the partial opening of the gate of the proteasome (PDB: 7DR7) [53].

#### 2.3.3. The PA28γ Regulator

A third member of the PA28 family is PA28γ, which is structurally homologous to PA28α and PA28β [88]. Unlike PA28α and PA28β, PA28γ is not inducible by IFNγ and is predominantly localized in the nucleus. This protein assembles into a ring-shaped homo-heptamer that binds to the α-rings of nuclear proteasomes and activates them (Figure 3C) [89,90]. In contrast to the PA28αβ regulator, which increases the catalytic activities of all active β-subunits, the PA28γ regulator was reported to selectively improve trypsin-like activity without affecting the other activities of the proteasome [88].

#### 2.3.4. The PA200 Regulator

The proteasome regulator PA200 is a 200 kDa nuclear protein that has a dome-like architecture (Figure 3D). Monomers of PA200 bind to one or both ends of the 20S proteasome and interact specifically with all α-subunits except α7 [91]. PA200 uses the C-terminal YYA (tyrosine–tyrosine–alanine) motif to induce rearrangements of the α-subunits of the 20S proteasome [92]. These rearrangements induce the partial opening of the proteasome gate possibly facilitating the entry of peptides or the exit of digestion products from the proteasome [91,92]. Moreover, recent structural data showed that PA200 has two openings surrounded by dense clusters of positively charged amino acids that are likely to be the gates of the PA200-20S proteasome (PDB: 6KWX and 6KWY) [92]. These positively charged amino acids that form the two openings of the PA200 could be accountable for the increased caspase-like activity of the PA200-20S proteasome [93,94]. PA200 RP is particularly abundant in the testes, and PA200-deficient mice are viable and exhibit no abnormalities except for a marked reduction in male fertility, suggesting an important role for the PA200 RP during spermatogenesis [95]. A recent study conducted by Javitt et al. showed that PA200 is upregulated in cancerous tissues, and its expression in non-small-cell lung carcinoma plays an anti-inflammatory role by attenuating IP activity [96]. Finally, upon exposure to ionizing radiation, proteasomes with 19S RP on one end and PA200 on the other end accumulate on chromatin, suggesting that it may play a role in double strand break repair [94].

## 3. The Differential Functions of the Proteasome Subtypes

The primary function of the proteasome is the turnover of damaged and misfolded proteins and the selective degradation of short-lived regulatory proteins. This function makes the proteasome a guardian of cellular integrity and a key regulator in numerous cellular functions. In addition to ubiquitin-controlled protein degradation, the proteasome can also degrade proteins bearing disordered domains in an ATP-independent and ubiquitin-independent-manner. In addition to protein degradation, the proteasome is also implicated in the production of antigenic peptides for MHC class I presentation. We will describe the mechanisms allowing the proteasome to perform these functions and the contribution of each proteasome subtype to these processes. Finally, we will detail the functions that are specific to the thymoproteasome and the immunoproteasome.

### 3.1. The Ubiquitin- and ATP-Dependent Proteasomal Degradation

Protein degradation is primarily facilitated by the ubiquitin proteasome system, which selectively tags undesirable proteins with ubiquitin moieties that target them for degradation by the 26S proteasome.

**Figure 4:**
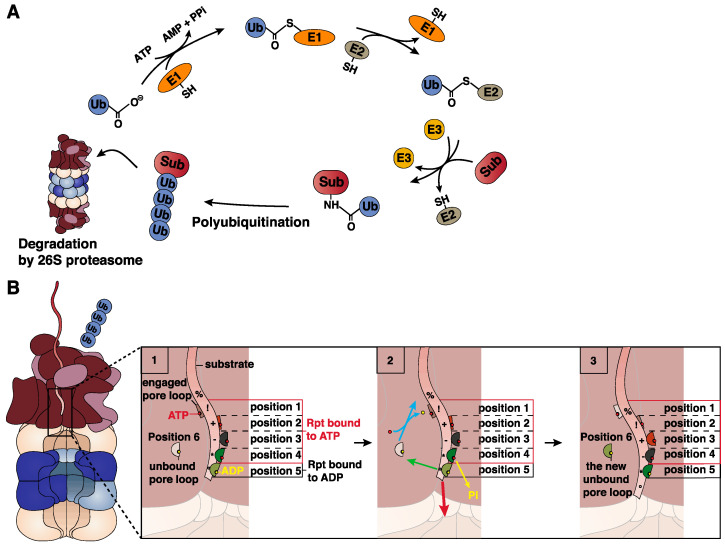
ATP- and ubiquitin-dependent degradation of proteins by the 26S proteasome. (**A**) Ubiquitin conjugation. Ubiquitin (Ub) conjugation requires the activity of three enzymes. E1 (a ubiquitin-activating enzyme) catalyses the ATP-dependent activation of the Ub moiety and its transfer to a conserved active cysteine on E1. Ub is then transferred to an active cysteine on E2 (a ubiquitin-conjugating enzyme). The transfer of the Ub moiety to the substrate requires the activity of an E3 enzyme (a ubiquitin ligase) that can interact with both E2 and the substrate. The resulting product is a protein–ubiquitin conjugate. Finally, the ubiquitin moiety already conjugated to the protein can undergo polymerization by the addition of ubiquitin moieties, forming a suitable tag that allows the targeting of the protein substrate for 26S proteasomal degradation. (**B**) Translocation of the protein substrate into the 26S proteasome. (1) Five of the six pore loops (presented here as half-circles) found in the AAA+ motor of the 19S regulatory particle align in a spiral staircase configuration entrapping the substrate. The four Rpt subunits with the pore loops at the uppermost positions are bound to an ATP molecule (red circle), and the Rpt subunit with the pore loop at the bottom position (position 5) is bound to an ADP molecule (yellow circle). The unbound pore loop occupies position 6 and is not bound to the substrate. (2) First, the Rpt subunit at position 4 hydrolyses the ATP molecule (yellow arrow), and the unbound Rpt subunit binds an ATP molecule. The newly formed Rpt-ATP subunit binds the substrate at the uppermost position as indicated by the light blue arrow, while the pore loop at position 5 disengages the substrate as indicated by the green arrow. Finally, the four Rpt subunits at positions 1–4, which are bound to the protein substrate, move downward, resulting in the translocation of the substrate (red arrow). (3) Following all these changes, the pore loop that was at position 6 occupies the uppermost position (position 1), the pore loop at position 3 occupies the penultimate position (position 4), the pore loop at position 4 occupies the bottom position (position 5), and the Rpt subunit with a pore loop at position 5 dissociates and becomes the new free pore loop at position 6 [104]. Symbols (%, !, +, -, * and °) were added on the protein substrate to aid in visualizing the translocation of the protein.

The ubiquitination of protein substrates is initiated by the covalent linkage of an ubiquitin protein through the creation of an isopeptide bond between the C-terminal glycine of the ubiquitin protein and the side chain amino group of a lysine residue in the substrate. Ubiquitination of the proteins targeted for 26S proteasome degradation is a process that requires the successive activity of the ubiquitin-activating enzyme (E1), the ubiquitin-conjugating enzyme (E2) and the ubiquitin ligase (E3) (Figure 4A). Additional ubiquitin moieties are then sequentially added to form a polymerized ubiquitin chain (Figure 4A), which induces the recruitment of protein substrates to the 26S proteasome through interaction of their ubiquitins either with 19S ubiquitin receptors Rpn1, Rpn10 and Rpn13 found at the periphery of the proteasome complex, or with several intracellular ubiquitin receptors that act as shuttles and transport substrates to the 19S RP [97,98,99]. In addition to the targeting signal provided by the ubiquitin molecules, proteins destined for degradation by the 26S proteasome should display an unstructured initiation signal of 34 to 44 amino acids long that allows them to access the pore loops of the 19S RP present at the centre of the AAA+ motor [100,101,102].

Recent high-resolution cryo-EM studies of the 26S proteasome elucidated the complex mechanism that leads to 26S proteasomal degradation of ubiquitinated protein substrates [103,104,105,106,107,108] (human 26S proteasome, PDB: 5T0C, 5T0G, 5T0H, 5T0I and 5T0J [103]; yeast 26S proteasome, PDB: 6EF0, 6EF1, 6EF2, 6EF3 and 5WVK [104]; yeast 26S proteasome, PDB: 3JCO and 3JCP [105]; yeast 26S proteasome, PDB: 4CR2 [107]; and yeast 26S proteasome, PDB: 5MP9, 5MPA, 5MAPB, 5MPC, 5MPD and 5MPE [108]). First, the unstructured initiation signal found in the protein substrate accesses the pore loops in the centre of the 19S RP. The 19S RP undergoes major conformational changes, which create a continuous central channel leading to the gate of the 20S proteasome and places the Rpn11 deubiquitinating enzyme directly above the central channel where it removes the ubiquitin chains [103,105,106,107,108]. After the substrate engagement, conformational switch and deubiquitination, the protein substrate unfolds and translocates into the 20S catalytic chamber. The mechanical unfolding and translocation of the protein substrate is driven by the ATP-dependent movements of the Rpt subunits that pull down the protein substrate closer to the catalytic chamber of the 20S proteasome (Figure 4B) [104]. Finally, the C-terminal HbYX motifs found in the Rpt2, Rpt3 and Rpt5 subunits bind the inter-subunit pockets of the outer α-ring, thus opening the gate of the 20S proteasome and allowing the entry of the unfolded substrate into the proteasome catalytic chamber [10,77,104,109]. For most 26S protein substrates, all catalytic subunits found in the catalytic chamber significantly contribute to proteolysis [110,111].

#### Proteasome Subtypes and the Degradation of Ubiquitinated Proteins

Studies have shown that the activity of the 19S RP is rate-limiting in the overall proteolysis of ubiquitinated proteins [112,113]. Indeed, using archaebacterial proteasomes, Benaroudj et al. found that the degradation of unfolded proteins by open-gated 20S proteasome mutants (with α-subunits lacking the N-terminal residues that block the entry of proteins into the 20S complex) was significantly more efficient than the degradation of the same substrates by the WT PAN (equivalent of the eukaryotic 19S regulator)-proteasome. These results confirmed that the activity of the regulator particle PAN slows down and therefore controls 26S proteasomal degradation [112]. Later, Henderson et al. confirmed by in vitro and in vivo assays using eukaryotic proteasomes that the 19S RP dictates the rate of 26S proteasomal degradation [113]. A recent study conducted by Bard et al. revealed the complete kinetics of 26S proteasomal degradation [114]. Using FRET-based assays, they showed that the substrate engagement step and the conformational switch are fast compared to the deubiquitination, translocation and unfolding steps. Moreover, they showed that the unfolding of 26S protein substrates is the rate-limiting step in the overall 26S proteasomal degradation [114]. Taken together, all these studies showed that protein degradation is more effective than protein delivery. Therefore, having different catalytic subunits inside the catalytic chamber should not affect the efficacy with which the different 26S proteasome subtypes degrade ubiquitinated proteins. In recent years, this matter was extremely controversial [115,116,117]. Seifert et al. showed that, in inflammatory conditions, up-regulation of the IP was associated with a gradual decrease in the amount of polyubiquitinated proteins that accumulate in response to IFNγ. They also showed that, in IFNγ-stimulated cells or in a mouse model of inflammatory disease (experimental encephalomyelitis), IP deficiency resulted in the accumulation of ubiquitinated proteins and aggresome-like inclusion bodies. Their in vitro experiment using purified 26S SP and 26S IP showed that the IP was more efficient at the degradation of an ubiquitinated substrate [117]. The authors suggested that a specific property of the immunoproteasome is to rapidly degrade newly synthesized ubiquitinated proteins, which accumulate in inflammatory conditions [117]. These observations were later disputed by Nathan et al., who showed, in a similar set of experiments, that the 26S SP had a similar capacity to the 26S IP for binding and degrading ubiquitinated protein substrates [116]. Moreover, they showed that the lack of IP in inflammatory conditions did not lead to the accumulation of ubiquitinated proteins [116]. We recently showed that, in non-inflammatory conditions, the 26S SP, 26S IP and the two 26S intermediate proteasomes degrade ubiquitinated proteins with the same efficacy [118]. The fact that the 26S SP and the 26S IP degrade proteins at a similar rate in non-inflammatory conditions is further supported by the observation that, in vivo, the overall levels of polyubiquitinated proteins between cells (splenocytes and bone-marrow derived macrophages) obtained from WT mice or mice deficient in one or the three immuno-subunits were similar [119,120]. Taken together, our results combined with results in the literature suggest that the turnover of ubiquitinated proteins in non-inflammatory conditions is a function performed equally well by the different 26S proteasome subtypes [118,119,120]. Whether the 26S IP is crucial to maintain protein homeostasis in inflammatory conditions remains controversial and requires further investigation.

### 3.2. The Ubiquitin- and ATP-Independent Proteasomal Degradation

In addition to the 26S proteasomal degradation heavily studied over the past years, evidence indicates that protein degradation can also be performed in an ATP- and ubiquitin-independent manner by the 20S proteasome [121,122]. Using an approach based on quantification by label-free nano LC-MS/MS of proteasome-interacting proteins, a recent study showed that, in nine human cell lines, more than 50% of the proteasomes available within cells exist in the free 20S proteasome form [123]. Moreover, biochemical analysis of rabbit reticulocyte lysates showed that free 20S proteasomes cleave more than 20% of all cellular proteins [124]. Studies performed using in vitro purified proteasomes showed that proteins featuring intrinsically disordered regions (IDR) can be substrates of the 20S proteasome [125,126,127,128]. The degradation of such proteins is more efficiently performed by the 20S proteasome than by the 26S proteasome [126]. The unstructured regions displayed by these proteins enable their entry into the catalytic chamber of the 20S proteasome in an ATP and ubiquitin-independent manner [122]. One of the mechanisms that help preventing their degradation by 20S proteasomes is their oligomerization into structurally stable complexes [125,129,130]. One example is the turnover of ornithine decarboxylase (ODC): monomeric ODC is an unstable protein rapidly degraded by the 20S proteasome, whereas functional homodimeric ODC resists 20S proteasomal degradation [129]. Studies have also shown that the two 20S proteasome interactors NAD(P)H quinone oxidoreductase 1 (NQO1) and DJ-1 can inhibit 20S proteasomal degradation by specifically binding the 20S proteasome, thus preventing substrate degradation [129,131,132]. Both NQO1 and DJ-1 have a conserved N-terminal region that consists of two positively charged residues followed by four hydrophobic residues. Moreover, these proteins adopt a Rossmann fold, composed of an extended parallel β-sheet surrounded by α-helices. A bioinformatic screen based on the shared characteristics found in NQO1 and DJ-1, revealed 15 additional proteins making a novel family of proteins called catalytic core regulators, which interact with the 20S proteasome and inhibit its function [133].

In addition to the degradation of proteins containing IDRs, the free 20S proteasome was shown to degrade oxidatively damaged proteins that can accumulate during oxidative stress [134,135]. Similarly to proteins containing IDRs, oxidized proteins have disordered regions that allow them to access the catalytic chamber of the free 20S proteasome [118,136]. Numerous biochemical studies have shown that under oxidative stress conditions, the 19S RP partly dissociates from the 20S proteasome with the help of protein Ecm29 and the heat shock protein 70 (Hsp70), thus increasing the proportion of free 20S proteasomes within cells [118,137,138,139]. After its dissociation, the free 19S RP is stabilized by the Hsp70 chaperone, which later helps in the reassembly of the 26S proteasome once the oxidative stress is eliminated [137]. In addition to the partial dissociation of the 26S proteasome, oxidative stress is associated with the depletion of 50% of cellular ATP [140,141,142]. Moreover, Inai et al. showed that an Rpn9-defective yeast strain with disrupted assembly of the 26S proteasome complex was more resistant to hydrogen-peroxide induced oxidative stress and was able to remove oxidatively damaged proteins more efficiently than wild type cells [143]. Shringarpure et al. showed that Chinese hamster and murine cell lines with compromised ubiquitin-conjugating activity degraded oxidized proteins at normal rates [144]. Altogether, studies showed that in oxidative stress conditions, the 26S proteasome activity is compromised and the free 20S proteasome is the main machinery that removes oxidatively damaged proteins [143,144,145].

Interestingly, free 20S proteasome complexes are also found in the serum and plasma and are significantly more abundant in the serum of patients with haematological malignancies, liver diseases or autoimmune disorders [146]. The mechanism leading to the release of extracellular 20S proteasomes in the cellular milieu is not yet clear but could result either from passive release from ruptured cells, active transport through secretory routes or exosome release [146]. Although extracellular 20S proteasomes might be involved in the clearance of disordered and oxidized proteins found in the extracellular space [146], their function is still largely unknown. Recently, it was suggested that degradation of osteopontin by extracellular 20S proteasomes could lead to the release of chemotactic peptides with potential implication in multiple sclerosis [147,148].

#### 3.2.1. Proteasome Subtypes and the ATP- and Ubiquitin-Independent Degradation of Proteins

Unlike ubiquitinated proteins, which are unfolded by the 19S RP before entering the catalytic chamber of the proteasome, proteins containing IDRs and oxidized proteins directly and passively access the catalytic chamber of the free 20S proteasome through their disordered regions, which are essential for the ATP- and ubiquitin-independent 20S proteasomal degradation [118,122,136]. As the entry of 20S protein substrates is not regulated by the 19S delivering system, the nature of the catalytic subunits found in the catalytic chamber of the 20S proteasome affects the efficacy with which the different 20S proteasome subtypes degrade these proteins. We recently evaluated the ability of the SP, SIP, DIP and IP to degrade oxidized proteins in a ubiquitin- and ATP-independent manner. Using purified 20S proteasomes, we showed that the three β5i-containing proteasomes, i.e., the IP and the two intermediate proteasomes, degrade oxidized calmodulin and oxidized haemoglobin faster than the SP. Similar to oxidized proteins, we showed that the intrinsically disordered tau was more efficiently degraded by the three β5i-containing proteasomes than by the SP [118]. Moreover, we showed that inhibiting the β5i catalytic subunit using the ONX-0914 inhibitor drastically reduced the capacity of the β5i-containing proteasomes to degrade oxidized proteins. Together, our results suggest that β5i-containing 20S proteasomes play an important role in the clearance of oxidized and intrinsically disordered proteins in an ATP- and ubiquitin-independent manner [118].

#### 3.2.2. Role of the Regulators in the ATP- and Ubiquitin-Independent Protein Degradation

In addition to the free 20S proteasome, the ATP- and ubiquitin-independent degradation of proteins could be carried out by proteasomes associated with regulatory particles, such as PA28αβ, PA28γ and PA200. Association of the 20S proteasome with PA28αβ enhances the ATP- and ubiquitin-independent degradation of oxidized proteins [149,150,151]. Moreover, we found an increased binding of PA28αβ to the 20S proteasome in oxidative stress conditions [118]. The role of the PA28αβ RP in the removal of oxidized proteins is further supported by the observation that PA28 is required for the removal of damaged proteins during embryonic stem cell fate specification [152]. New structural data using hydrogen-deuterium eXchange coupled to mass spectrometry (HDX-MS) revealed a higher protection from deuteration in the PA28αβ-SP complex as compared to PA28αβ-IP, suggesting a higher interaction of PA28αβ with SP [153]. However, using mass spectrometry-based label-free quantitative proteomics on affinity-purified proteasome complexes, Fabre et al. showed a preferential binding of PA28αβ to the IP and the other two β5i-containing proteasomes [154]. This could reinforce the activity of the β5i-containing proteasomes towards oxidatively damaged proteins. The PA28γ-bound proteasomes are responsible for the ATP- and ubiquitin-independent degradation of several nuclear protein substrates, such as the steroid receptor coactivator 3; the cell cycle inhibitors p21, p16 and p19; and the transcription factor MAFA [155,156,157]. Apart from these specific substrates, PA28γ was also shown to enhance the ATP- and ubiquitin-independent degradation of oxidized proteins [150]. Moreover, we showed an increased binding of PA28γ to the 20S proteasome in oxidative stress conditions, thus supporting its potential role in the removal of oxidatively damaged proteins [118]. Unlike PA28αβ and PA28γ, PA200 reduced the ability of 20S proteasomes to degrade oxidized proteins [150]. The PA200-proteasome also mediates the ATP- and ubiquitin-independent degradation of some unstructured substrates and acetylated histones [158,159,160].

### 3.3. Production of MHC Class I Peptides

**Figure 5:**
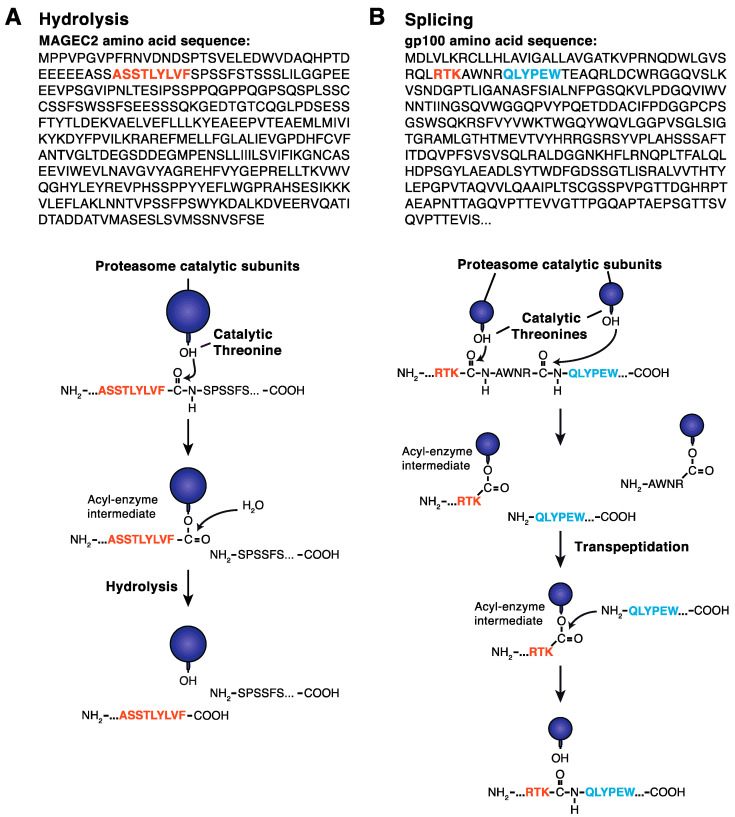
(**A**) Peptide bond hydrolysis. The hydroxyl group of the N-terminal threonine of the proteasome catalytic subunits attacks the carbonyl group of the peptide bond. This leads to the production of an acyl-enzyme intermediate in which the carbonyl group of the peptide fragment remains attached to the hydroxyl group of the N-terminal threonine of the proteasome by an ester link. To release the peptide fragment, a water molecule present in the catalytic chamber of the proteasome attacks the ester link between the peptide and the threonine residue, restoring the hydroxyl group of the catalytic threonine and producing the C-terminal end of the peptide. (**B**) Peptide splicing by the proteasome. The splicing of antigenic peptide RTK_QLYPEW derived from the gp100 is shown. Following formation of the acyl-enzyme intermediate involving the fragment RTK and the hydroxyl group of the N-terminal threonine of the proteasome, the free N-terminal amino-group of peptide QLYPEW present in the proteasome chamber attacks the acyl-enzyme intermediate, leading to the formation of the peptide RTK_QLYPEW composed of two peptide fragments originally distant in the protein.

Once a protein reaches the catalytic chamber of the 20S proteasome, it is cleaved by the six catalytically active β-subunits. Mutagenesis and structural analyses performed on archaebacterial and eukaryotic proteasomes revealed that the hydroxyl group of the N-terminal threonine (Thr1) found in all catalytic subunits initiates the peptide bond hydrolysis of the protein substrate (Figure 5A) [161,162]. The products of proteasomal degradation are peptides, which can range from 2 to 24 residues in length. The resulting peptides are either further degraded by intracellular peptidases into amino acids or transported to the lumen of the ER, where they are loaded onto MHC class I molecules with the help of the peptide loading complex (PLC). MHC-class I peptide complexes are then transported to the cell surface, where they play an essential role in immunosurveillance [163,164]. Early studies reported that presentation of the model peptide ovalbumin was dependent on ubiquitination and that proteasome inhibitors blocked the production of peptides for MHC class I presentation, providing strong evidence for the role of the proteasome in the production of peptides binding to MHC class I molecules [163,165,166,167,168]. The use of proteasome inhibitors also suggested that the proteasome is responsible for the production of the C-terminus of class I peptides but is not required for the generation of their N-terminus, which is, instead, produced by non-proteasomal aminopeptidases, such as ERAPs (endoplasmic reticulum aminopeptidases) [169,170]. In healthy cells, those peptides originate from normal autologous proteins, and thus fail to activate the immune system.

#### 3.3.1. Proteasome Subtypes and the Production of Canonical MHC Class I Peptides

As the different proteasome subtypes comprise specific catalytic subunits that endow them with distinct cleavage properties (Figure 2), they can shape the peptide repertoire presented by MHC class I molecules [59,120,171,172,173]. In particular, the IP displays increased chymotrypsin-like and trypsin-like activities, suggesting that the IP favours the production of peptides with a hydrophobic or basic C-terminus, which are well suited for binding to MHC class I molecules [51,52]. In line with this, studying splenocytes isolated from mice deficient in all three immuno-subunits, Kincaid et al. observed a decrease in MHC class I surface expression, which was suggested to result from an impaired export of mature MHC class I complexes to the cell surface [120]. These mice poorly presented most MHC class I epitopes tested, and the class I peptidome of their splenocytes was significantly different from that of wild-type splenocytes [120]. Another study showed that the major changes that were observed in the MHC class I peptide repertoire upon IFNγ stimulation were not attributable to changes in the proteome, suggesting a major role of IP induced by IFNγ [174]. In line with this, a recent study on organoids derived from colorectal cancer patients showed a qualitative change in the immunopeptidome upon IFNγ treatment [175]. This change in the peptide repertoire is caused by the occurrence of not only peptides derived from IFNγ-induced genes but also peptides generated by enzymes displaying a chymotrypsin-like activity. Since the IFNγ inducible IP has an increased chymotrypsin-like activity, these peptides could be the result of IP processing [175]. In cell lines derived from melanoma patients, the induction of the IP was associated with a higher detection of neoantigens and tumour-associated antigens by autologous tumour-infiltrating lymphocytes (TILs) [176]. In cellular assays, TILs derived from these patients and directed toward specific antigens showed a higher reactivity toward cells overexpressing IP. Moreover, the expression of IP in the patient tumours correlated with a better response to checkpoint inhibitor therapy [176]. In line with the changes in the peptide repertoire associated with IP expression, several antigenic peptides recognized by CTL on tumours were also shown to be more efficiently produced by the IP when compared to the SP [172,177]. Taken together, these studies show that a change in the peptide repertoire occurs upon IP expression. It was suggested that by displaying a different peptide repertoire on the cell surface upon inflammation, the IP could focus CTL responses on IP-dependent epitopes, which are not present in non-inflamed tissues, and could therefore contribute to protection against autoimmune diseases [178].

Nevertheless, it is also clear that a number of antigenic peptides are better produced by the SP than by the IP. The first example of such a peptide was described by Morel et al., who studied a CTL clone isolated from a renal cell carcinoma patient and was shown to derive from the ubiquitous protein RU1 [172]. Surprisingly, despite ubiquitous expression of the RU1 protein, this peptide is expressed on kidney cancer cells but not on the autologous EBV-B cells [172]. This was explained by the fact that the peptide is processed by the SP, which is found in the cancer cells, but not by the IP, which is expressed by the EBV-B cells. Since then, a number of other peptides produced by the SP and not by the IP were subsequently identified. The analysis of in vitro proteasome digests indicated that the lack of production of the peptide by one proteasome subtype generally resulted from the occurrence of a major destructive cleavage inside the antigenic peptide [59,171,177]. In line with this conclusion, the inhibition of subunit β1 or β5 rescued processing by the SP of the IP-dependent antigenic peptides suggesting the degradation of these peptides by the SP [179]. Due to their particular cleavage properties, intermediate proteasomes were also shown to produce a unique repertoire of peptides, with the HLA-A2-restricted peptides MAGE-A10_254–262_ and MAGE-C2_191–200_ being exclusively produced by the DIP and HLA-A2-restricted peptide MAGE-A3_271–279_ being processed by the SIP [59,171]. Intermediate proteasomes consequently enlarge the MHC class I repertoire to include peptides that can be processed by both dendritic cells (containing IP and intermediate proteasomes) and tumour cells (containing SP and intermediate proteasomes). Antigenic peptides produced by intermediate proteasomes might therefore represent valuable targets for cancer immunotherapy [4].

#### 3.3.2. Proteasome Subtypes and the Production of Spliced Peptides

A few years ago, we and others showed that the proteasome could also produce antigenic peptides by peptide splicing, i.e., through the creation of a new peptide bond between two peptide fragments originally distant in the parental protein [180,181]. The peptide splicing reaction takes place inside the catalytic chamber of the proteasome and occurs by a transpeptidation involving an acyl-enzyme intermediate formed between the C-terminus of a peptide fragment and the hydroxyl group of the N-terminal Thr1 of the catalytic β-subunit. This acyl-enzyme intermediate undergoes a nucleophilic attack by the N-terminus of another peptide present in the proteasome chamber. This transpeptidation restores the hydroxyl group of the Thr1 and releases a spliced peptide made of two noncontiguous fragments (Figure 5B) [181]. Thus far, six peptides produced by peptide splicing have been formally identified using T cells isolated from cancer patients [180,181,182,183,184,185]. Mass-spectrometry-based approaches were also used to attempt to identify additional spliced candidates; however, due to the complexity of the technique and the lack of biological evidence confirming the existence of the identified spliced peptides, this remains a matter of intense debate [186,187,188,189,190,191]. Among the six spliced peptides originally identified using anti-tumour T cells, four were composed of two non-contiguous peptide fragments that were spliced in the reverse order to that in which they appear in the parental protein [182,183,184,185]. The comparison of the production of spliced peptides by the SP and the IP revealed that some peptides were better produced by the SP and others better produced by the IP [192]. This demonstrated that both proteasome subtypes are intrinsically able to splice peptides, in agreement with the transpeptidation model. The efficacy of one proteasome subtype to produce a given spliced peptide appears to depend on its ability to perform the cleavages required to release the peptide fragments involved in the splicing reaction. Additionally, the splicing of a given peptide will also depend on the occurrence of destructive cleavages limiting the availability of the splicing partners. Affinity of the attacking peptide fragment for the primed binding site of the catalytic subunit could also play a role. Although the production of spliced peptides by the intermediate proteasomes or the thymoproteasome has not yet been investigated, it is expected that these proteasomes would also be able to splice peptides.

### 3.4. Thymoproteasome and Positive Selection

As a result of its unique cleavage activity, the thymoproteasome is associated with a unique MHC class I peptidome: comparing mouse embryonic fibroblast (MEF) cells expressing either the IP or the thymoproteasome, a difference of about 30% in their peptide repertoire was detected [173]. As the thymoproteasome is specifically expressed in cTECs, it is rational to suggest that it plays a role in the positive selection of progenitor T cells. Mice deficient in catalytic subunit β5t have a reduced number of splenic CTLs, a modified T-cell receptor repertoire and an altered T-cell responsiveness. These mice have no defects in CD4^+^ T cells and no problems in the negative selection of autoreactive T cells, supporting the notion that the thymoproteasome is essential for the positive selection of CD8^+^ T cells [14,193,194]. The role of the thymoproteasome was further examined using β5t transgenic mice in a β5i^−/−^ background [195]. These mice express thymoproteasome in the thymic cortex, in the thymic medulla and in the periphery in all cells that ordinarily express the IP. In these mice, the cTEC-mediated positive selection was not affected, however CD8^+^ T cells were less abundant compared to the control mice. This study shows that having a restricted expression of thymoproteasome in the thymic cortex is crucial for an efficient development of CD8^+^ T cells possibly because it allows a change in the peptide repertoire between the thymic cortex and medulla, thus preventing enhanced negative selection [195]. Alternatively, the thymoproteasome could also contribute to positive selection by producing peptides that have biochemical or structural properties promoting the positive selection of CD8^+^ T cells [196].

### 3.5. Role of the IP in Immune-Related Pathways

Aside from its role in MHC class I presentation, the IP plays a role in a number of other immune-related pathways, which makes IP inhibition a rational strategy to treat autoimmune diseases, inflammatory bowel disease and certain cancers or to prevent transplant rejection [197,198,199,200,201,202,203,204,205]. Indeed, IP inhibition attenuates disease progression in several murine models of autoimmune diseases, such as diabetes, colitis and arthritis [206,207,208]. In line with this, in a mouse model of colitis, knocking-out or inhibiting β5i suppressed the expansion of pro-inflammatory T helper 1 and T helper 17 cells due to decreased STAT1 and STAT3 phosphorylation, while it promoted the differentiation of regulatory T cells following an increase in SMAD phosphorylation [206]. Moreover, in a mouse model of rheumatoid arthritis, specific IP inhibition by LMP7 inhibitor ONX-0914 limited the production of pro-inflammatory cytokines interleukin (IL)-2, IFNγ and TNF by T cells and of pro-inflammatory IL-23 by CD14^+^ monocytes [207]. This inhibition of IL-23 production was shown to occur following the apoptosis of IP-expressing monocytes, which are the main producers of this cytokine. Apoptosis was preceded by the accumulation of polyubiquitinylated proteins and the induction of an unfolded protein response, and could be prevented by inhibitors of protein translation, showing that the IP plays a crucial role in the maintenance of protein turnover in human CD14^+^ monocytes [209]. Additionally, T cells knocked-out for the immuno-subunits showed reduced survival and proliferation following their transfer in virus-infected mice, suggesting that IP is also required for T cell expansion [210,211]. As the IP is only expressed in a limited number of cell types and tissues, several studies are currently exploring the beneficial effects of inhibiting immuno-subunits to induce proteotoxic stress in malignant cells that predominantly express the IP, such as leukemias and myelomas [36,212,213].

## 4. Conclusions

In summary, proteasomes are responsible for the selective degradation of damaged and misfolded proteins, as well as the turnover of short-lived regulatory proteins. Proteasomal degradation can take place in an ATP- and ubiquitin-dependent manner. This process of degradation requires the activity of the 26S proteasome, which consist of the 20S proteasome bound to the 19S regulatory particle. Moreover, the proteasome can also degrade proteins that have disordered regions, such as oxidized proteins or proteins bearing intrinsically disordered regions. This mechanism of degradation is ATP- and ubiquitin-independent and is performed either by the free 20S proteasome or by the 20S proteasome linked to regulatory particles, such as PA28αβ, PA28γ or PA200. Peptides produced following proteasomal degradation can bind MHC class I molecules, making the proteasome an important player in allowing the immune system to continuously survey the emergence of abnormal cells. Proteasomes found in mammalian cells exist under different proteasome subtypes differing in the combination of catalytic subunits found in their catalytic chamber: the SP, the IP, the two intermediate proteasomes SIP and DIP and the thymoproteasome. The spermatoproteasome contains an alternative α4 subunit named α4s and is found in male germ cells, where it plays a key role in spermatogenesis. Although studies have shown that the SP, the IP and the SIP and DIP are equally efficient at degrading ubiquitinated proteins, they differ in other functions. For instance, the IP plays a crucial role in immune cells by promoting a proinflammatory environment. Moreover, β5i-containing proteasomes are more efficient than the SP in the ATP- and ubiquitin-independent degradation of oxidized proteins and proteins containing intrinsically disordered regions. Finally, because they display different catalytic activities that influence the set of antigenic peptides they produce, the different proteasome subtypes shape the repertoire of peptides presented at the cell surface by MHC class I molecules.

## Data Availability

Not applicable.
